# A dissociation between consolidated perceptual learning and sensory adaptation in vision

**DOI:** 10.1038/srep38819

**Published:** 2016-12-16

**Authors:** Nitzan Censor, Hila Harris, Dov Sagi

**Affiliations:** 1 School of Psychological Sciences and Sagol School of Neuroscience, Tel Aviv University, Tel Aviv 69978, Israel; 2 Department of Neurobiology, Weizmann Institute of Science, Rehovot 76100, Israel

## Abstract

Perceptual learning refers to improvement in perception thresholds with practice, however, extended training sessions show reduced performance during training, interfering with learning. These effects were taken to indicate a tight link between sensory adaptation and learning. Here we show a dissociation between adaptation and consolidated learning. Participants trained with a texture discrimination task, in which visual processing time is limited by a temporal target-to-mask window defined as the Stimulus-Onset-Asynchrony (SOA). An initial training phase, previously shown to produce efficient learning, was followed by training structures with varying numbers of SOAs. Largest interference with learning was found in structures containing the largest SOA density, when SOA was gradually decreased. When SOAs were largely kept unchanged, learning was effective. All training structures yielded the same within-session performance reduction, as expected from sensory adaptation. The results point to a dissociation between within-day effects, which depend on the number of trials per se regardless of their temporal structure, and consolidation effects observed on the following day, which were mediated by the temporal structure of practice. These results add a new dimension to consolidation in perceptual learning, suggesting that the degree of its effectiveness depends on variations in temporal properties of the visual stimuli.

There is now ample evidence showing that adult visual sensitivity can improve with repeated practice^1^, through perceptual learning^2^. These learning processes have been attributed to memory consolidation^3^,^4^ mechanisms. Thus, following initial encoding of the visual memory, it undergoes stabilization or enhancement evident as offline gains in performance^2^,^5^,^6^,^7^. In addition, while perceptual learning is based on repeated training, previous studies have shown that over-exposure to the task results in performance deterioration^8^,^9^,^10^, possibly involving adaptation-related mechanisms^11^,^12^,^13^. Thus, such previous studies have challenged the traditional view according to which ‘practice makes perfect’, showing that increasing task exposure can reduce performance, and interfere with consolidation of efficient learning mechanisms.

Here we set to determine the link between the temporal structure of training, and performance changes within and between training sessions. We used the texture discrimination task (TDT)^5^. In this task, observers have to determine whether an array of diagonal bars embedded in a background of horizontal bars, is horizontal or vertical. To challenge visual processing, a patterned mask is presented following the task target frame. The target-to-mask stimulus onset asynchrony (SOA) can be varied within the session to obtain a psychometric curve from which the SOA discrimination threshold is derived. Indeed, this represents daily-life situations in which visual stimuli are available for limited processing times. In a previous study^14^ we showed that an initial training session of ~200 trials per target location, but not of ~800 trials, improves SOA threshold the next day. Here we tested how different temporal structures in the training regime above 200 trials affect acquisition and consolidation of learning. Temporal variations were induced by increasing the number of SOAs presented in a structured gradually decreasing order following an initial threshold measurement, testing conditions ranging from a fixed SOA to 11 SOAs. Thus, after obtaining a baseline threshold (200 trials), we varied the SOA-driven temporal structures within the training session, and re-tested sensitivity thresholds at the end of the training session, and on the following day to assess consolidation strength. We report a dissociation between within-day effects which depend on the number of trials per se regardless of their temporal structure, and consolidation effects observed on the following day which were mediated by the temporal structures following practice. These results suggest that the effectiveness of consolidation is reduced with structured variations in temporal properties of the visual stimuli.

## Methods

### Subjects

The subjects were 24 paid undergraduate students with normal or corrected-to-normal vision. Due to possible effects of prior experience, all of the experiments were performed by naïve subjects. All subjects started the experiments with no prior experience in the task. All subjects gave their written informed consent. The work was carried out in accordance with the Code of Ethics of the World Medical Association (Declaration of Helsinki), and approved by the local Institutional Review Board of the Weizmann Institute.

### Apparatus

The stimuli were presented on a 19′′ Mitsubishi Diamond Pro 930SB color monitor, using a PC with an Intel Pentium processor. The luminance of the stimulus (line textures) was 64 cd/m^2^ in an otherwise dark environment.

### Stimuli and Task

**Figure 1:**
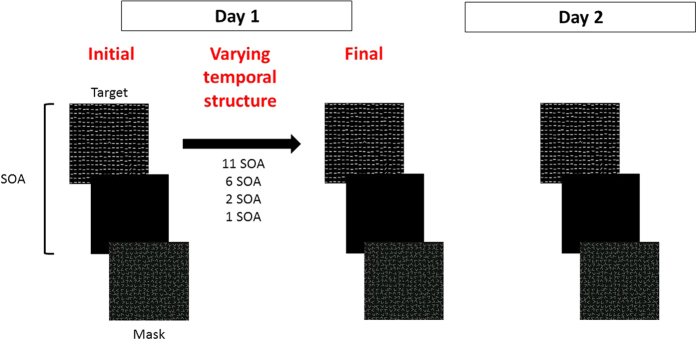
Texture discrimination with varying temporal structures. Participants performed the texture discrimination task, in which a target frame is followed by a patterned mask, limiting processing time (stimulus onset asynchrony, SOA). The task requires to discriminate whether an array of 3 diagonal bars embedded in a background of horizontal bars, was horizontal or vertical. Following an initial threshold measurement, the temporal structure was varied, before the final Day 1 threshold measurement. On Day 2 all subjects performed an identical threshold measurement (see methods).

The standard texture stimuli was used^5^,^9^, consisting of a target frame, which appeared for 40 ms. The target was followed by a patterned mask which appeared for 100 ms, as shown in Fig. 1. Observers had to decide whether an array of 3 diagonal bars embedded in a background of horizontal bars (19 × 19, 0.45° × 0.03° each, and spaced 0.74° apart) was horizontal or vertical. Display size was 14° by 14° of visual angle, viewed from a distance of 100 cm. The target appeared randomly and equally, either in the upper left or lower right visual quadrant (for the 11 SOAs group the targets appeared either in the lower left or lower right visual quadrant) 4.46°–6° of visual angle from center of display. Fixation was enforced by a forced-choice letter-discrimination task, between a “T” and an “L”, at the center of the display. The time-interval between the target stimulus and the mask (stimulus-to-mask onset asynchrony, SOA) was manipulated. After an initial SOA wherein above 90% correct texture discrimination occurred was determined, the SOA was gradually decreased by SOA-dependent steps of 20–40 ms (340, 300, 260, 220, 200, 180, 160, 140, 120, 100, 80 ms) of 3–4 blocks of trials per SOA (unless stated otherwise, see the ‘random SOA’ group below). Blocks contained 12 trials (6 trials per target location, ~200 per target location per session, 30–60 minutes, initial and final thresholds measurements on day 1) or 50 trials (~800 trials per target location per session, 90–150 minutes, day 2). Each psychometric curve obtained was fitted with the Weibull function, with an additional finger error parameter **1-p**, yielding the function detailed in equation (1):


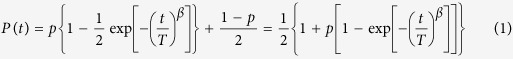


where **T** is the threshold for each curve, defined as the SOA for which 81.6% of responses were correct. In each session, the threshold SOA for both target locations was averaged. Sessions were terminated when the subject reached an SOA with close to chance level of performance (defined as less than 65% correct responses).

### Experimental procedures

The observers were randomly assigned to different experimental groups. On day 1 all groups underwent identical short initial and final threshold measurements (~200 trials per target location as detailed above), with different temporal structures introduced in between during two sub-sessions (~200 trials each), 12 subjects with 1 or 2 SOAs, and 9 subjects with multiple SOAs, as follows:

~11 SOAs (similar to the initial threshold measurement described above, n = 6), 6 SOAs (300, 260, 220, 180, 140, 100 ms, n = 3), 2 SOAs (260, 160 ms, n = 3), 1 SOA (either 340 ms, n = 3; 120, 140, or 160 ms, n = 3; or above threshold SOA, 140, 160, or 180 ms, n = 3). When more than 1 SOA was presented, the SOAs were presented in a decreasing order starting from the highest, 18 trials per SOA (at each location). When the lowest SOA was reached, trials started again decreasing from the highest SOA, and so on until the sub-session ended at ~200 trials. Of note, additional 3 subjects performed the 2 sub-sessions with SOAs presented randomly (340, 300, 260, 220, 200, 180, 160, 140, 120, 100, 80 ms).

On day 2, all subjects performed a single identical long session (~800 trials per target location per session, as detailed above).

## Results

**Figure 2:**
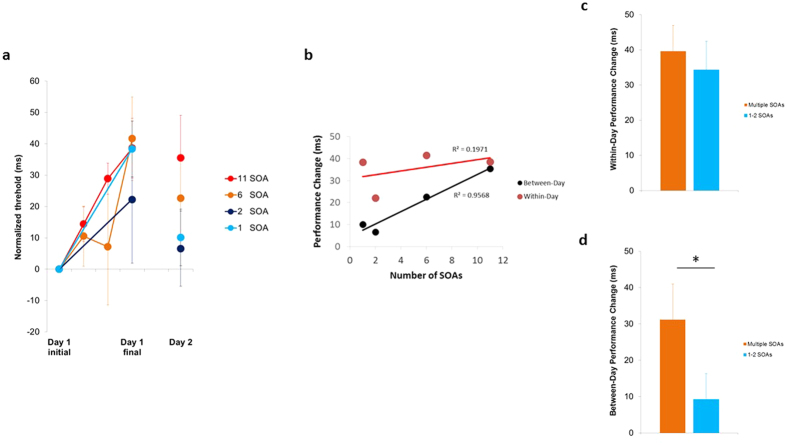
A dissociation between within-day and between-day performance changes. **(a)** Normalized discrimination thresholds for each training condition. **(b)** All groups showed similar within-day deterioration (day 1 final-initial thresholds), however performance differentiated following consolidation (day 2): Increasing the number of SOAs interfered with between-day consolidation, resulting in increased day 2 discrimination thresholds (relative to day 1 initial thresholds). **(c)** Within-day changes were highly consistent across multiple and 1 or 2 SOAs. **(d)** Between-day changes were significantly impaired with multiple SOAs relative to 1 or 2 SOAs. *P < 0.05. Error bars are s.e.m.

On day 1, all groups had very similar initial and final thresholds, with SOA-driven temporal structures introduced in-between. An ANOVA showed that initial thresholds were similar across all groups (mean 130 ± 4 ms s.e., F_3,17_ = 2.37, P = 0.11). Figure 2a displays the day 1 and day 2 thresholds for all groups relative to their initial threshold (including within-session threshold derivations in the varying temporal structure phase, when there are enough SOAs to allow it). Results showed that for all groups (Fig. 2b), the thresholds elevated with additional training, with all groups exhibiting remarkably similar within-day threshold elevation, or performance deterioration (37 ± 5 ms, P < 0.0005). Of note, these within-day changes were consistent across multiple, and 1 or 2 SOAs (Fig. 2c). This indicates that these drops in performance are determined by the total amount of trials within a daily session (which was comparable for all groups), rather than the temporal structures (that varied across groups during between the initial and final thresholds measurements).

On the other hand, the number of ordered SOAs predicted day 2 performance (Pearson’s correlation r = 0.98, P < 0.03, Fig. 2b). In contrast to the within-day effects, the between-days performance changes varied based on the temporal structures following day 1 initial threshold measurement. Thus, increasing the number of ordered SOAs interfered with between-day consolidation, resulting in increased day 2 discrimination thresholds (relative to day 1 initial thresholds). Indeed, between-day changes with multiple SOAs (n = 9, 31 ± 10 ms, P < 0.03, corrected for multiple comparisons) were significantly impaired (P < 0.05) relative to 1 or 2 SOAs (n = 12, 9 ± 7 ms, P = 0.44) (Fig. 2c). Interestingly, preliminary results show that when day 1 SOAs were randomized between the initial and final measurements, with the highest number of SOAs, there were substantial within-day decrements (37 ± 10 ms, n = 3), however between-day consolidation was not impaired (day 2 thresholds relative to day 1 initial thresholds 5 ± 15 ms, P = 0.79, n = 3), although more experiments are required to substantiate the later observation (see Discussion).

## Discussion

Taken together, the results show that inducing structured temporal variations impacts between-day performance changes. Increasing the number of SOAs strongly reduces performance measured on the following day. A dissociation was observed between within-day effects, which depend on the number of trials per se regardless of their temporal structure, and consolidation effects observed on the following day which were mediated by the temporal structures following practice.

This dissociation fits with a model according to which online processes resulting in perceptual deterioration accumulate with repeated stimulus or task exposure. Previous studies have suggested that such processes are mediated by adaptation-like mechanisms in early visual networks. On the other hand, between-session learning relies on readout mechanisms facilitated by higher-order networks that encode a template for correct discrimination^11^,^15^. Importantly, the current state of the template always corresponds to past experience, thus, templates are expected to reduce efficiency when stimulus conditions are changed. In accordance with earlier results showing that our task involves temporal learning^16^,^17^, the present results suggest that the template update can reduce temporal sensitivity, thus reducing consolidation efficiency. Accordingly, not only is the gradual SOA variation a critical parameter, but also the continuous gradual transitions, resulting in small but consistent changes in SOA to which the learning mechanism shows reduced sensitivity. These temporal conditions are less prominent when reducing the number of SOAs thus increasing the SOA differences, and, having more trials at each SOA, allowing the template to remain tuned to the SOAs encountered. In such cases, consecutive SOAs are similar, and the present stimulation matches past stimulation, thus the template update does not alter sensitivity. This account predicts intact consolidation when the SOAs are presented randomly, since here, as with fixed SOA, the past matches the present and the template is not updated maladaptively. Indeed this is implied in our additional experiment, although more experiments are required to test this prediction.

Since day 2 training is intense, and gradual, performance may indicate how the template of learning deals with restricted training conditions. Worse performance on day 2 may indicate ineffective learning following day 1 training, while better performance may indicate an efficient template that is able to deal well with restrictive training conditions^14^. Accordingly, the inefficient template update during gradual stimulus variation, as noted above, may therefore result in noisy connections, limiting performance^18^.

In sum, we show that incremental stimulus variations induce interference and by that reduces the effectiveness of consolidation in perceptual learning. This adds a new temporal dimension to consolidation in visual perceptual learning, commonly relating to the spatial dimensions relevant to visual cortex architecture.

## Additional Information

**How to cite this article**: Censor, N. *et al*. A dissociation between consolidated perceptual learning and sensory adaptation in vision. *Sci. Rep.* **6**, 38819; doi: 10.1038/srep38819 (2016).

**Publisher's note:** Springer Nature remains neutral with regard to jurisdictional claims in published maps and institutional affiliations.
